# Sinais de Injúria Cardíaca em Pacientes Pediátricos com COVID-19 Gravemente Enfermos: Uma Experiência de Centro Único no Brasil

**DOI:** 10.36660/abc.20210200

**Published:** 2022-05-04

**Authors:** Marcelo Felipe Kozak, Yuri Caldas Pessoa, Luciana Oliveira Castro e Silva, Manuela Baima Cabral, Barbara Costalonga Pereira Leite, Juliana Duarte Diniz, Aline Saliba, Selma Harue Kawahara

**Affiliations:** 1 Hospital da Criança de Brasília, José de Alencar Brasília DF Brasil Hospital da Criança de Brasília, José de Alencar, Brasília, DF – Brasil

**Keywords:** COVID-19, Coração, Criança

## Abstract

**Fundamento:**

Alguns pacientes com COVID-19 apresentam injúria miocárdica.

**Objetivo:**

Detectar a injúria miocárdica em pacientes criticamente doentes, e comparar o envolvimento cardíaco entre crianças com síndrome respiratória aguda grave (SARS) e crianças com síndrome inflamatória multissistêmica (MIS-C).

**Métodos:**

Todas as crianças acometidas da COVID-19 admitidas em uma unidade de terapia intensiva de referência foram cadastradas de forma prospectiva e fizeram uma ecografia transtorácica bidimensional (ETT-2D) e um teste de troponina I cardíaca (cTnI) nas primeiras 72 horas. Para a análise estatística, um p <0,05 bilateral foi considerado significativo.

**Resultados:**

33 pacientes foram incluídos, dos quais 51,5% apresentaram cTnI elevada e/ou ETT-2D anormal e 36,4% precisaram de suporte cardiovascular, que foi mais frequente em pacientes com cTnI elevada e anormalidades em ETT-2D do que em pacientes com exames normais (83,3% e 33,3%, respectivamente; p 0,006, 95% IC = 0,15-0,73). Os achados de ETT-2D mais comuns foram efusão pericárdica (15,2%) e regurgitação tricúspide/mitral (15,2%). Sinais de envolvimento cardíaco foram mais comuns na MIS-C que na SARS. Pacientes com MIS-C também apresentaram um índice mais alto de necessidade de suporte cardiovascular (66,7% X 25%, p 0,03, 95% IC = -0,7 a -0,04) e um índice mais frequente de cTnI elevada (77,8% X 20,8%; p 0,002, 95% IC = 0,19 a 0,79). Os valores preditivos negativos de cTnI para detecção de anormalidades de ETT-2D foram 100% para pacientes com MIS-C, e 73,7% para pacientes com SARS.

**Conclusão:**

Sinais de injúria cardíaca foram comuns, especialmente em pacientes com MIS-C. As anormalidades na ETT-2D foram sutis. A realização de um teste de cTnI na admissão pode ajudar os prestadores de assistência de saúde a discriminar os pacientes com uma necessidade mais urgente de uma ETT-2D.

## Introdução

Até o início de fevereiro de 2021, o número de casos da COVID-19 no mundo já havia alcançado mais de 105 milhões de pessoas, incluindo quase 9,5 milhões no Brasil, dos quais 231 mil morreram.^1^ Em relação ao tropismo viral, os pulmões não são o único alvo da COVID-19. O comprometimento cardiovascular em pacientes infectados pela COVID-19 já foi bem descrito mundialmente. A infecção SARS-COV2 já foi associada a injúria miocárdica aguda, miocardite, arritmias e tromboembolismo venoso. Essas doenças predispõem os pacientes a doenças graves e morte, especialmente aqueles com doenças cardiovasculares pré-existentes.^2 , 3^

Crianças têm sido relatadas como uma pequena fração dos casos confirmados de COVID-19, representando cerca de 2% do número de pacientes hospitalizados com SARS (síndrome respiratória aguda grave), e cerca de 0,5% do número de mortes no Brasil e em outros países.^1 , 4^ No início da pandemia, a maioria das crianças infectadas pelo novo coronavírus SARS-COV2 era assintomática ou apresentava sintomas leves. Posteriormente, observou-se um aumento no número de crianças internadas em unidades de terapia intensiva pediátricas (UTIP) em todo o mundo com choque na presença da infecção por SARS-CoV-2.^5 - 9^ Eles apresentaram uma síndrome hiperinflamatória com manifestações semelhantes às da doença de Kawasaki, síndrome do choque tóxico, ou linfohistiocitose hemofagocítica secundária. Essa condição foi chamada de “síndrome inflamatória multissistêmica em crianças” (MIS-C), e foi comumente associada a disfunção cardíaca, hipotensão, arritmias e dilatação da artéria coronária.^10 - 13^

Os principais objetivos deste estudo foram detectar sinais de injúria miocárdica em pacientes pediátricos criticamente doentes com COVID-19 internados em uma UTIP de referência no Brasil, por meio de teste de troponina I cardíaca (cTnI) e de ecografia transtorácica bidimensional (ETT-2D), e comparar o envolvimento cardíaco entre crianças com SARS e crianças com MIS.

## Métodos

Este foi um estudo coorte de centro único observacional realizado em um hospital pediátrico terciário, escolhido como o único centro de referência para pacientes pediátricos com COVID-19 criticamente doentes no Distrito Federal, Brasil, uma área com uma população aproximada de 3,5 milhões. O estudo foi aprovado pelo comitê de Ética local, que dispensou a necessidade de consentimento (protocolo CAAE 34511120.0.0000.8927). O projeto foi desenhado em maio de 2020, antes da primeira admissão de um paciente pediátrico em nossa UTIP. Todas as crianças com COVID-19 confirmada admitidos na UTIP entre 28 de maio e 27 de agosto de 2020, que apresentavam SARS ou MIS-C, eram incluídas no estudo de forma prospectiva. A confirmação da COVID-19 era feita por RT-PCR (reação em cadeia da polimerase em tempo real) de amostras de swabs nasofaríngeos ou orofaríngeos, ou por testes imunológicos para imunoglobulina M ou imunoglobulina G para glicoproteína spike viral usando um ensaio de imunoabsorção enzimática (ELISA).

O protocolo do estudo determinou que todos os pacientes da COVID-19 admitidos na UTIP fossem submetidos a uma ETT-2D e um teste cTnI nas primeiras 72 horas de internação.

Os exames de ETT-2D eram realizados no leito por três cardiologistas pediátricos experientes, de acordo com as diretrizes da Sociedade Americana de Ecocardiografia, usando um Toshiba Xario SSA 660-A (Toshiba Medical Systems Corporation, Japão).^14^ Os seguintes parâmetros e estruturas de ETT-2D foram avaliados: função sistólica do ventrículo esquerdo (VE) (usando o método de Teichholz), função sistólica do ventrículo direito (VD) (usando observação visual e excursão sistólica do plano anular tricúspide), movimento da parede, função valvar (usando Doppler a cores), pericárdio, artérias coronárias (seus diâmetros foram indexados à superfície corporal e diagramados em relação a escores z), e sinais de hipertensão pulmonar (pressão sistólica do VD > 40 mmHg ou pressão arterial pulmonar média > 25 mmHg).^15 , 16^ Uma FEVE <55% foi considerada disfunção sistólica do VD. A disfunção sistólica do VD era considerada quando o escore z da TAPSE era <-2 ou por análise qualitativa.^17^ Alguns pacientes foram submetidos a mais de um ETT-2D durante sua internação, de acordo com sua evolução clínica e a critério do médico assistente, mas apenas o ETT-2D realizado na admissão foi considerado para a análise.

A medição da cTnI foi realizada usando o kit Elecsys Troponin I STAT (Roche Diagnostics), que tinha um valor de corte de 0,1 ng/ml.

Foram descritos os dados demográficos do paciente, o tipo de apresentação da COVID-19, doenças pré-existentes, duração da internação na UTI, duração da internação hospitalar, tipo de suporte respiratório, duração do uso do suporte respiratório, necessidade de suporte vasoinotrópico, escore vasoinotrópico máximo (VIS), cTnI inicial e de pico, e anormalidades de ETT-2D no dia da admissão. Em alguns casos, testes pró-BNP (peptídeo natriurético cerebral) e CKMB (creatinofosfoquinase MB) foram pedidos pelos médicos assistentes, para avaliação clínica. Os resultados também foram apresentados.

A definição de SARS esteve de acordo com a do Centro para controle e prevenção de doenças ( *Center for Disease Control and Prevention* - CDC), que a define como uma síndrome respiratória aguda grave associada ao diagnóstico da infecção por SARS-CoV-2. A definição de MIS-C também esteve de acordo com a do CDC, que a define como uma doença grave que leva à hospitalização pacientes com menos de 21 anos de idade, com febre por pelo menos 24 horas, apresentando evidência laboratorial de inflamação, com envolvimento multissistêmico de órgãos (≥ 2), infecção por SARS-CoV-2 confirmada ou presumida, e nenhum diagnóstico alternativo plausível.^18^

### Análise estatística

Para a avaliação da normalidade dos dados, foi realizado o teste de Kolmogorov-Smirnov para calcular a área abaixo da curva normal, presumido como sendo quando aproximadamente 95% da área estava dentro de 1,96 de desvio padrão da média. As variáveis contínuas foram expressas como média ± desvio padrão (DP) ou mediana e faixa interquartil, de acordo com a existência de dados normais. As variáveis categóricas foram descritas como porcentagens. O teste Mann-Whitney foi usado para comparar variáveis contínuas a distribuições distorcidas. Para comparar variáveis contínuas com distribuição normal, foi usado o teste t de Student não pareado. O teste de Kruskal-Wallis, com o método de Conover-Iman, foi usado para comparar variâncias de VIS entre pacientes com combinações diferentes de achados em exames. Os valores preditivos positivos e negativos de cTnI para a detecção de anormalidades de ETT-2D foram calculados. Um p <0,05 bilateral foi considerado estatisticamente significativo. Todas as análises foram realizadas utilizando o programa StatsDirect, v. 3.3.4 2020 (Merseyside, Reino Unido).

## Resultados

Trinta e três pacientes foram incluídos no estudo, com idade que variou de 31 dias a 17 anos de idade. Quando o último paciente foi incluído, a população de nosso estudo representava 3% de todas as crianças diagnosticadas com COVID-19 em nossa região, o que significa que praticamente todas as crianças criticamente doentes em nossa área devem ter sido incluídas no estudo.^19^ A maioria (72,7%) apresentava diagnóstico de SARS. Havia três pacientes com doenças cardíacas congênitas: um em pós-operatório de Senning (derivação atrial para transposição corrigida das grandes artérias), outro com um defeito do septo atrioventricular total, e um terceiro com comunicação interatrial, diagnosticado durante o estudo. Houve uma morte devido a uma complicação por celulite periorbitária em um paciente com aplasia de medula óssea. Esse paciente havia apresentado um diagnóstico de SARS. A Tabela 1 resume os achados gerais de nosso estudo.

**Tabela 1:** – Características demográficas e gerais de pacientes pediátricos com COVID-19

Características	Todos os pacientes (33)	SARS (24)	MIS-C (9)	p	95% IC
Masculino	19 (57,6%)	15 (62,5%)	4 (44,4%)	0,35	
Idade (anos)	6,4 ± 5,6	5,7 ± 5,6	8,2 ± 5,5	0,2	
IMC (Kg/m^2^)	18,2 ± 6	17,9 ± 5,8	18,9 ± 6,7	0,79	
**Doenças crônicas**	**18 (54,5%)**	**16 (66,7%)**	**2 (22,2%)**	**0,02**	**0,06 a 0,69**
Hematológicas	6 (18,2%)	6 (25%)	0		
Cardíacas	3 (9,1%)	2 (8,3%)	1 (11,1%)		
Neurológicas	3 (9,1%)	2 (8,3%)	0		
Obesidade	3 (9,1%)	2 (8,3%)	1 (11,1%)		
Nefrológicas	2 (6,1%)	2 (8,3%)	0		
Pulmonares	2 (6,1%)	2 (8,3%)	0		
Diabetes	1 (3%)	1 (4,2%)	0		
**Duração na internação na UTI (dias)**	**6 (3-12)**	**7 (3-12)**	**5 (3-7)**	**0,32**	
Duração da internação hospitalar (dias)	14 (10-19)	15,5 (12-21.2)	10 (7-14)	0,09	
**Suporte respiratório**	**27 (81,8%)**	**22 (91,7%)**	**5 (55,6%)**	**0,008**	**0,05 a 0,67**
Apenas oxigênio	14 (42,4%)	13 (54,2%)	1 (11,1%)		
CPAP	1 (3%)	1 (4,2%)	0		
Ventilação mecânica	12 (36,4%)	8 (33,3%)	4 (44,4%)		
**Duração do suporte respiratório (dias)**	**5 (1-9)**	**6 (2,5-10,5)**	**2 (0-5)**	**0,14**	

*IMC: índice de massa corporal; UTI: unidade de terapia intensiva; CPAP: pressão positiva contínua nas vias aéreas.*

### Injúria cardíaca e comprometimento cardiovascular

Dezessete dos 33 pacientes (51,5%) apresentaram cTnI elevada e/ou ETT-2D anormal. Cinco pacientes apresentaram cTnI elevada e ETT-2D anormal (15,2%); cTnI elevada isolada foi identificada em sete pacientes (21,2%); e ETT-2D anormal isolada foi identificada em cinco pacientes (15,2%).

Doze pacientes (36,4) necessitaram de suporte cardiovascular com drogas inotrópicas ou vasoativas, selecionadas e tituladas de acordo com os achados hemodinâmicos e a critério da equipe da UTIP. Dez desses 12 pacientes (83,3%) tinham cTnI e/ou ETT-2D anormal, enquanto entre os 21 pacientes que não precisaram de suporte cardiovascular, sete (33,3%) apresentaram exames anormais. Essa diferença foi estatisticamente significativa (p = 0,006, 95% IC = 0,15-0,73).

Entre os 5 pacientes com cTnI elevada e ETT-2D anormal (a), 80% precisaram de drogas inotrópicas ou vasoativas (VIS 26 ± 24,8); entre os pacientes com ETT-2D anormal isolada (b), 60% precisaram dessas drogas (VIS 5 ± 5), entre os pacientes com cTnI elevada isolada (c), 42,8% precisaram de drogas inotrópicas ou vasoativas (VIS 8,4 ± 13,4), enquanto o uso dessas drogas foi identificado em 12,5% dos pacientes com exames normais (d) (VIS 1,2 ± 3,6). Em relação ao VIS, detectou-se uma diferença estatisticamente significativa apenas entre pacientes “a” e “d” (p= 0,006); as diferenças entre outros pares comparados não eram significativas (a X b, p= 0,23; a X c, p= 0,14; b X c, p= 0,83; b X d, p= 0,15; c X d, p= 0,18). A Tabela 2 resume os achados cardiovasculares de nossa população.

**Tabela 2:** – Suporte cardiovascular e achados cardíacos de pacientes pediátricos com COVID-19

Características	Todos os pacientes (33)	SARS (24)	MIS-C (9)	p	95% IC
Suporte cardiovascular	12 (36,4%)	6 (24%)	6 (66,7%)	0,03	-0,69 a -0,04
VIS máximo *	19 ± 17	11 ± 6	28 ± 21	0,051	-37,6 a 2,6
**cTnI anormal**	**12 (36,4%)**	**5 (20,8%)**	**7 (77,8%)**	**0,002**	**-0,79 a -0,19**
cTnI de pico		0,41 ± 0,48	0,61 ± 0,61	0,56	
Eco e cTnI anormais		1/5 (20%)	4/7 (57,1%)	0,2	
Pró-BNP alto	22/25 (88%)	15/17 (88,2%)	7/8 (87,5%)	0,96	
CKMB alto	10/19 (52,6%)	5/11 (45,4%)	5/8 (62,5%)	0,46	
**ETT-2D anormal**	**8 (24,2%)**	**4 (16%)**	**4 (44,4%)**	**0,08**	
Disfunção sistólica do VE	2 (6,1%)	0	2 (22,2%)	0,1	
Disfunção sistólica do VD	1 (3%)	1 (4,2%)	0	0,28	
RT/RM	5 (15,2%)	2 (8,3%)	3 (33,3%)	0,47	
Efusão pericárdica	5 (15,2%)	4 (16,7%)	1 (11,1%)	0,03	0,04 a 0,96
AMP	1 (3%)	0	1 (11,1%)	0,28	
Anormalidades coronárias	0	0	0		
Escore z de ACEP	-0,23 ± 0,8	-0,23 ± 0,88	-0,23 ± 0,57	1	
Escore z de ACD	0,16 ± 0,87	0,14 ± 0,88	0,22 ± 0,9	0,81	

ETT-2D: ecografia transtorácica bidimensional; BNP: peptídeo natriurético cerebral; CKMB: creatinofosfoquinase MB; cTnI: troponina I cardíaca; VIS: escore vasoinotrópico; VE: ventrículo esquerdo; ACEP: artéria coronária esquerda principal; RM: regurgitação mitral; ACD: artéria coronária direita; VD: ventrículo direito; RT: regurgitação tricúspide; AMP: anormalidade no movimento da parede. *Entre os que precisaram de suporte cardiovascular

Anormalidades em ETT-2D foram encontradas em 10 pacientes (30,3%). Os achados mais comuns foram efusão pericárdica leve (5 pacientes) e regurgitação tricúspide/mitral não trivial (5 pacientes). Apenas dois pacientes apresentaram disfunção sistólica do VE, totalmente recuperada ao receber alta hospitalar. Um dos pacientes com disfunção sistólica do VE também apresentou anormalidade de contração segmentar e efusão pericárdica leve. Esse paciente fez uma ressonância magnética cardíaca (RMC) 7 meses mais tarde, e a efusão pericárdica ainda estava presente, sem outros sinais de miocardite. Não foi detectado nenhum caso de dilatação coronária ou hipertensão pulmonar. A Tabela 3 descreve as principais características dos 10 pacientes que apresentaram ETT-2D anormal. O paciente número 3, um menino em pós-operatório de Senning, apresentou SARS e flutter atrial. Esse paciente havia realizado uma ETT-2D em outra instituição 50 dias antes do diagnóstico de COVID-19. Naquele momento, tanto a regurgitação tricúspide quanto a disfunção sistólica do VD foram consideradas leves, diferente do que se observou na admissão.

**Tabela 3:** – Achados clínicos, laboratoriais e ecocardiográficos dos oito pacientes que apresentaram ETT-2D anormal na admissão

Número do paciente	1	2	3	4	5	6	7	8	9	10
Sexo	M	M	M	F	F	F	M	F	F	F
Idade	14 a	12 a	7 a	13 a	3 a	11 a	20 m	10 m	2 a	13 a
ASC	1,21	1,39	1,02	1,55	0,54	1,15	0,58	0,41	0,35	1,58
IMC	13,1	17,2	17,7	22,6	12,3	15,5	14,7	16,7	16,6	17,7
Comorbidade	MSC	-	DCC	-	BMA	-	-	-	DPOC e DP	Diabetes
Apresentação	SARS	MIS-C	SARS	MIS-C	SARS	MIS-C	MIS-C	SARS	SARS	SARS
Duração na internação na UTI (dias)	12	5	22	7	8	6	3	2	12	5
Duração na internação hospitalar (dias)	16	10	34	9	8	10	7	15	18	8
Suporte respiratório (dias)	4	5	10	2	8	3	0	2	17	5
Ventilação mecânica (dias)	0	3	2	0	1	2	0	0	0	2
CTnI de pico (corte 0,1 ng/ml)	<0,1	0,27	<0,1	1,91	<0,1	0,58	0,22	0,13	<0,1	<0,1
Normalização de cTnI	-	3 dias	-	4 dias	-	5 dias	1 dia	1 dia	-	-
VIS máximo	0	50	5	15	10	55	10	0	10	0
Pró-BNP (corte 125 pg/ml)	NA	27183	5546	5341	988	22985	31188	1133	2445	NA
CKMB (corte 25 ng/ml)	NA	19,4	17,9	28,4	NA	41	23,6	24,4	NA	NA
ETT inicial	EP mínima	RM leve	Disfunção grave do VD, disfunção leve/moderada do VE, RT grave	Disfunção leve do VE, anormalidade no movimento da parede, EP leve	EP leve	RM moderada, disfunção leve do VE	RT leve	EP moderada	EP leve	RM leve
ETT na alta	NA	normal	Disfunção grave do VD, disfunção leve do VE, RT moderada	Normal	EP leve		RT leve	RM leve, EP mínima	NA	NA

*BMA: aplasia de medula óssea; BNP: peptídeo natriurético cerebral; DCC: doença cardíaca congênita; CKMB: creatinofosfoquinase MB; PC: paralisia cerebral; DPOC: doença pulmonar obstrutiva crônica; VE: ventrículo esquerdo; RM: regurgitação mitral; NA: não disponível; EP: efusão pericárdica; VD: ventrículo direito; MSC: anemia falciforme; VIS: escore vasoativo inotrópico.*

### MIS-C X SARS

A necessidade do suporte cardiovascular foi mais frequente em pacientes que apresentaram MIS-C do que nos que apresentaram SARS (66,7% e 25%, respectivamente, p= 0,03, 95% IC = -0,7 a -0,04). Entre os que precisaram de suporte cardiovascular, o escore VIS era mais alto nos pacientes com MIS-C do que nos pacientes com SARS, mas esse achado não foi estatisticamente significativo (28,2 ± 21,3 para MIS-C e 10,7 ± 5,7 para SARS, p= 0,1).

A cTnI elevada foi observada com mais frequência em pacientes com MIS-C do que em pacientes com SARS (77,8% e 20,8%, respectivamente; p= 0,002, 95% IC = 0,19 a 0,79); entretanto, a diferença na cTnI de pico não foi estatisticamente significativa (p= 0,19). Foram encontradas anormalidades de ETT-2D em 44,4% dos pacientes com MIS-C e em 25% dos pacientes com SARS (p= 0,28). A única diferença estatisticamente significativa na frequência de anormalidades de ETT-2D foi a taxa de de disfunção sistólica do VE, que era mais comum nos pacientes com MIS-C do que nos pacientes com SARS (22,2% X zero; p= 0,02, 95% IC = -0,06 a 0,55). Não houve diferenças em relação à duração da internação na UTI (p= 0,58), duração da internação hospitalar (p= 0,86), e duração do suporte respiratório (p= 0,61).

Os valores preditivos positivos de cTnI para detecção de anormalidades de ETT-2D foram de 70% para pacientes com MIS-C, e de 20% para pacientes com SARS. Os valores preditivos negativos foram, respectivamente, 100% e 73,7%. A dosagem de pró-BNP não foi realizada em todos os pacientes, já que não fazia parte do protocolo, mas pró-BNP elevado foi um achado comum quando realizado (91,7%), independentemente do tipo de COVID apresentado pelo paciente. A dosagem de CKMB não foi realizada em todos os pacientes, já que não fazia parte do protocolo, mas esteve elevada em 52,6% dos pacientes, sem diferença significativa de acordo com a apresentação da COVID-19.

## Discussão

Este estudo foi desenvolvido com o objetivo de detectar sinais de injúria miocárdica em pacientes pediátricos criticamente doentes, e comparar o envolvimento cardíaco entre crianças com SARS e crianças com MIS-C. Os métodos diagnósticos escolhidos para essa avaliação foram a ecocardiografia transtorácica e a dosagem de troponina I cardíaca. Nossa UTIP era a única unidade de referência para casos pediátricos atendidos pelo SUS em nossa região. Como esse estudo foi desenhado antes da admissão do primeiro caso, foi possível incluir todas as crianças criticamente doentes com COVID-19 em nossa área geográfica durante a primeira onda da COVID-19 no Brasil em 2020. Esse é, possivelmente, o ponto mais importante de nosso estudo.

O envolvimento miocárdico na COVID-19 é comum e se manifesta histologicamente de formas diferentes: miocardite-like, inflamação miocárdica, doença tromboembólica e infarto. Esses achados têm sido corroborados por estudos de ressonância magnética cardíaca em pacientes adultos e pediátricos e por evidências patológicas.^8 , 20 - 24^ Entretanto, esses métodos para se diagnosticar miocardite, edema miocárdico ou injúria cardíaca isquêmica não são viáveis na maioria das crianças, devido ao caráter invasivo da biópsia endomiocárdica e às dificuldades em se realizar RMC em crianças gravemente doentes, especialmente quando havia restrições quanto ao uso de técnicas de imagem avançadas durante a pandemia da COVID-19.

A cTnI é uma proteína contrátil cardíaca específica, encontrada em cardiomiócitos, que tem alta sensibilidade (95%) para o diagnóstico de miocardite viral em crianças.^25^ Entretanto, ela também pode ser liberada em casos de stress excessivo da parede, isquemia miocárdica ou aumento da demanda de oxigênio do miocárdio, situações frequentemente encontradas em pacientes com COVID-19, especialmente naqueles com problemas médicos crônicos.^26^ Uma das ressalvas sobre a medição de cTnI em crianças é que os valores de corte têm o objetivo de diagnosticar infartos em adultos, sendo que esses valores podem estar relacionados à quantidade de tecido lesionado.^27^ Portanto, é razoável argumentar que, se estamos usando valores de corte de adultos, a detecção de troponina cardíaca elevada em crianças pode revelar uma lesão maior ao coração. Mesmo em adultos com COVID-19, exames de RMC anormais têm sido encontrados sem uma elevação de troponina cardíaca simultânea.^20^ Apesar disso, alguns autores têm relatado sua experiência com pacientes com MIS-C, demonstrando que a elevação da troponina é um achado comum, ocorrendo em mais de 70% dos casos.^6 , 7 , 28^ Em nosso estudo, aproximadamente

50% dos pacientes apresentaram cTnI elevada e/ou anormalidades ecocardiográficas, confirmando o alto índice de injúria miocárdica nesse subgrupo de pacientes.

Um achado muito relevante em nosso trabalho é o fato de que pacientes que tiveram tanto cTnI elevada quanto ETT-2D anormal precisaram de mais suporte cardiovascular do que os que apresentaram exames normais, o que está de acordo com alguns estudos na população adulta com COVID-19 e SARS. Esses estudos descrevem que a evolução clínica é pior nos casos em que há extravasamento de troponina e ETT-2D anormal.^29 - 31^ Em nosso estudo coorte, isso foi representado por um índice mais alto de pacientes que necessitaram de drogas inotrópicas ou vasoativas, como também em doses mais altas.

Anormalidades em ETT-2D foram encontradas em 30,3% de nossa população. Os achados mais comuns foram efusão pericárdica leve e regurgitação tricúspide/mitral leve. Apenas dois pacientes apresentaram disfunção sistólica do VE, totalmente recuperada no momento da alta hospitalar. As anormalidades da ETT-2D encontradas em nosso estudo foram majoritariamente transitórias e seguidas da normalização da cTnI, revelando a evolução dinâmica da doença e, possivelmente, um processo de cura.

Os dois pacientes com disfunção sistólica do VE representaram apenas 16,7% dos pacientes que precisaram inotrópicos e vasopressores em nosso estudo, sugerindo que essa doença tem um caráter bastante vasoplégico ou inflamatório, ao contrário de um estado de síndrome de débito cardíaco baixo. Um desses pacientes com disfunção sistólica do VE teve o pico mais alto de cTnI durante a realização deste estudo. Em relação à função sistólica do VE, vários achados já foram descritos por outros autores: Grimaud e Ramcharam identificaram uma queda na fração de ejeção do VE em mais de 80% de seus pacientes com MIS-C admitidos em suas UTIP em choque.^6 , 32^ Essas diferenças podem estar relacionadas a políticas locais de admissão na UTI, tempos diferentes de diagnósticos, características diferentes dos pacientes (características demográficas, presença de comorbidades); estrutura genética diferente dos pacientes com interações diferentes com o SARS-COV2, causando respostas imunes distintas; cepas diferentes do vírus causando graus diferentes de comprometimento cardiovascular; metodologias de estudo diferentes (momento dos exames por imagem, escolha do método de avaliação da função cardíaca, etc.).

A cTnI elevada foi encontrada em aproximadamente 80% dos pacientes com MIS-C e em aproximadamente 20% dos pacientes com SARS; entretanto, a cTnI elevada não foi associada nem à disfunção sistólica do VE, nem ao choque circulatório. Cinco dos sete pacientes (71,4%) com cTnI elevada no subgrupo com MIS-C apresentaram alguma anormalidade em ETT-2D, enquanto, no subgrupo com SARS, esse índice foi de 20% (1/5). É importante notar que, na população deste estudo, um teste de cTnI normal na admissão tinha um valor preditivo negativo

alto, sugerindo que esse teste poderia ser usado para descartar a injúria cardíaca, evitando exames de imagem cardiovascular em pacientes selecionados. Embora o número de pacientes com cTnI elevada seja pequeno, ainda assim parece haver um possível benefício em se realizar o teste de cTnI como teste de triagem para anormalidades na ETT-2D. Além disso, foi identificado que a elevação da cTnI e a presença de achados anormais na ETT-2D foram transitórios, com um comportamento clínico de uma miocardite aguda comum não fulminante. Nesse contexto, o tempo para o diagnóstico é essencial, embora o impacto clínico desse diagnóstico ainda seja desconhecido. Em relação à miocardite típica, a incidência de cardiomiopatia crônica no futuro dessas crianças ainda não é conhecida.

Nenhum dos pacientes apresentou anormalidades coronárias, o que está de acordo com os achados de Grimaud et al., na França.^32^ Outros estudos também identificaram um baixo índice de anormalidades coronárias.^7 , 33^ Outros achados de imagem da COVID-19 em crianças também foram descritos. No estudo de Ramcharan et al., 67% dos pacientes apresentaram regurgitação valvar temporária.^6^ No estudo de Grimaud et al., 65% dos pacientes apresentaram regurgitação tricúspide ou mitral não trivial, e a efusão pericárdica foi observada em 40% de sua casuística.^32^ Encontramos regurgitação tricúspide ou mitral não trivial em 15% dos pacientes de nosso estudo coorte, o mesmo índice de efusão pericárdica, sem diferenciação entre SARS e MIS-C.

Alguns estudos também relataram mudanças no ritmo cardíaco em pacientes com manifestações sistêmicas da infecção por SARS-COV2. Em um estudo coorte realizado em Nova Iorque com 393 pacientes, observou-se que 17,7% dos pacientes hospitalizados com SARS-COV2 e em uso de ventilação mecânica tinham arritmias, em comparação com 1,9% daqueles que não precisaram de ventilação mecânica.^34^ Em outro estudo coorte na China, com 187 pacientes, observou-se que 5,9% dos pacientes tinham taquiarritmias enquanto estavam hospitalizados.^29^ O único caso de arritmia observado neste estudo foi o de uma criança em pós-operatório de Senning, uma situação em que as arritmias atriais são conhecidas como complicações tardias da cirurgia. Nesse caso, não se tem certeza se a arritmia foi causada pela COVID-19. Esse paciente apresentou uma piora na função sistólica do VD, com cTnI normal.

### Limitações do estudo

Este estudo foi desenhado em maio, quase simultaneamente com o anúncio do fenótipo da MIS-C da COVID-19. Portanto, não tínhamos informações suficientes sobre a MIS-C naquele momento, e o desenho do estudo não incluiu a avaliação de marcadores inflamatórios ou de coagulopatia, embora alguns pacientes tenham sido submetidos a esses exames. Não analisamos o quadro clínico hemodinâmico (pressão arterial, tempo de enchimento capilar, frequência cardíaca, lactato sanguíneo) dos pacientes, e o uso de

drogas vasoativas ou inotrópicas ficou a critério do médico assistente. Optamos por usar o escore VIS para representar a gravidade do comprometimento cardiovascular de maneira padrão. Não avaliamos parâmetros de deformação miocárdica e admitimos que mudanças na deformação miocárdica podem estar presentes antes da queda da fração de ejeção.

## Conclusões

A prevalência de sinais de injúria miocárdica em crianças infectadas pela COVID-19 que necessitaram de cuidado intensivo foi alta (50%), e isso não foi exclusividade dos pacientes com MIS-C. Pacientes com MIS-C com troponina I cardíaca elevada e achados anormais em ETT-2D muito frequentemente apresentam sinais de choque. Os marcadores de injúria cardíaca foram temporários e os resultados iniciais, em geral, foram favoráveis. Por último, considerando o alto número de pacientes infectados admitidos em UTIP em todo o mundo e que os recursos de atendimento de saúde podem ser limitados, a realização de um teste de cTnI pode ajudar os prestadores de assistência de saúde a discriminar os pacientes com uma necessidade mais urgente de uma ETT-2D.
