# Supplementary material for: Sinais de Injúria Cardíaca em Pacientes Pediátricos com COVID-19 Gravemente Enfermos: Uma Experiência de Centro Único no Brasil
Source: Arq Bras Cardiol. 2022 May 4;118(5):937–45. [Article in Portuguese] doi: 10.36660/abc.20210200 (PMC9368871; doi:10.36660/abc.20210200)
Supplement: Translation

# Translation: Signs of Cardiac Injury in Critically Ill Paediatric Patients with COVID-19: a Single-Center Experience in Brazil

**Authors:** Marcelo Felipe Kozak, Yuri Caldas Pessoa, Luciana Oliveira Castro e Silva, Manuela Baima Cabral, Barbara Costalonga Pereira Leite, Juliana Duarte Diniz, Aline Saliba, Selma Harue Kawahara

**Affiliations:** 1 Brasil Hospital da Criança de Brasília, José de Alencar, Brasília, DF – Brasil

**Keywords:** COVID-19, Heart, Child

## Abstract

**Background:**

Some patients with COVID-19 present myocardial injury.

**Objective:**

To detect myocardial injury in critically ill paediatric patients, and to compare cardiac involvement between children with severe acute respiratory syndrome (SARS) and children with multisystemic inflammatory syndrome (MIS-C).

**Methods:**

All COVID-19 children admitted to a referral intensive care unit were prospectively enrolled and had a two-dimensional echocardiogram (2D-TTE) and a cardiac troponin I (cTnI) assay within the first 72 hours. For statistical analysis, two-sided p < 0.05 was considered significant.

**Results:**

Thirty-three patients were included, of which 51.5% presented elevated cTnI and/or abnormal 2D-TTE and 36.4% needed cardiovascular support, which was more frequent in patients with both raised cTnI and 2D-TTE abnormalities than in patients with normal exams (83.3% and 33.3%, respectively; p 0.006, 95% CI = 0.15-0.73). The most common 2D-TTE findings were pericardial effusion (15.2%) and mitral/tricuspid regurgitation (15.2%). Signs of cardiac involvement were more common in MIS-C than in SARS. MIS-C patients also presented a higher rate of the need for cardiovascular support (66.7% vs 25%, p 0.03, 95% CI = -0.7 to -0.04) and a more frequent rate of raised cTnI (77.8% vs 20.8%; p 0.002, 95% CI = 0.19 to 0.79). The negative predictive values of cTnI for the detection of 2D-TTE abnormalities were 100% for MIS-C patients and 73.7% for SARS patients.

**Conclusion:**

signs of cardiac injury were common, mainly in MIS-C patients. 2D-TTE abnormalities were subtle. To perform a cTnI assay upon admission might help providers to discriminate those patients with a more urgent need for a 2D-TTE.

## Introduction

Until early February 2021, the number of cases of COVID-19 in the world had already reached more than 105 million people, including nearly 9.5 million in Brazil, of whom 231,000 died.^1^ As for viral tropism, lungs are not the only COVID-19 target. Cardiovascular compromise in COVID-19 infected patients has been well described worldwide. SARS-COV2 infection has been linked to acute myocardial injury, myocarditis, arrhythmias, and venous thromboembolism. These conditions predispose patients to severe disease and death, mainly those with pre-existing cardiovascular diseases.^2 , 3^

Children have been reported as a small fraction of confirmed COVID-19 patients, representing around 2% of the number of hospitalized patients with SARS (severe acute respiratory syndrome) and around 0.5% of the number of deaths in Brazil and other countries.^1 , 4^ At the beginning of the pandemic, most of the children infected by the new coronavirus SARS-COV2 were asymptomatic or presented mild symptoms. Later, an increasing number of children admitted to paediatric intensive care units (PICU´s) was observed worldwide, with shock in the presence of SARS-CoV-2 infection.^5 - 9^ They presented with a hyperinflammatory syndrome with manifestations similar to Kawasaki’s disease, toxic shock syndrome or secondary hemophagocytic lymphohistiocytosis. This condition was named “Multisystemic Inflammatory Syndrome in Children” (MIS-C), and has been commonly associated with cardiac dysfunction, hypotension, arrhythmias and coronary artery dilatation.^10 - 13^

The main objectives of this study were to detect signs of myocardial injury in critically ill paediatric patients with COVID-19 admitted to a referral PICU in Brazil, through cardiac troponin I (cTnI) assay and two-dimensional transthoracic echocardiogram (2D-TTE), and to compare cardiac involvement between children with SARS and children with MIS.

## Methods

This was an observational single-center cohort study performed at a tertiary children´s hospital, elected as the only referral center for critically ill paediatric COVID-19 patients in Distrito Federal, Brazil, an area with an estimated population of 3.5 million. This study was approved by the local Research Ethic Committee, which waived the need for consent (protocol CAAE 34511120.0.0000.8927). The project was designed in May 2020, before the first admission of a paediatric patient at our PICU. All children with confirmed COVID-19 admitted in the PICU between May 28^th^and August 27^th^, 2020, presenting either with SARS or MIS-C, were prospectively included in the study. The confirmation of COVID-19 was made either by using real time polymerase chain reaction (RT-PCR) from samples of nasopharyngeal or oropharyngeal swabs, or by immunological testing for Immunoglobulin M or Immunoglobulin G to viral spike glycoprotein using an Enzyme-Linked Immunosorbent Assay (ELISA).

The study protocol determined that all COVID-19 patients admitted to the PICU underwent a 2D-TTE and a cTnI assay within 72 hours of hospital admission.

The 2D-TTE exams were carried out at bedside by three experienced paediatric cardiologists, following the guidelines of the American Society of Echocardiography, using a Toshiba Xario SSA 660-A (Toshiba Medical Systems Corporation, Japan).^14^ The following 2D-TTE parameters and structures were assessed: left ventricular (LV) systolic function (using the Teichholz method), right ventricular (RV) systolic function (using eye-ball and tricuspid annular plane systolic excursion), wall motion, valvular function (using color Doppler), pericardium, coronary arteries (their diameters were indexed to body surface and plotted against Z-scores), and signs of pulmonary hypertension (RV systolic pressure > 40 mmHg or mean pulmonary artery pressure > 25 mmHg).^15 , 16^ A LVEF < 55% was considered to be an LV systolic dysfunction. RV systolic dysfunction was considered when TAPSE Z-score was < -2 or by qualitative analysis.^17^ Some patients underwent more than one 2D-TTE during their hospitalization, according to their clinical course and at the discretion of the assistant physician, but only the 2D-TTE performed upon admission was considered for analysis.

The measurement of cTnI was performed using the Elecsys Troponin I STAT kit (Roche Diagnostics), which had a cut-off value of 0.1 ng/ml.

The present study described the patient’s demographic data, type of COVID-19 presentation, pre-existing conditions, length of ICU stay, length of hospital stay, type of respiratory support, length of respiratory support, need for vasoinotropic support, maximum vasoinotropic score (VIS), initial and peak cTnI and 2D-TTE abnormalities on the admission day. In some cases, pro-BNP (brain natriuretic peptide) and CKMB (creatine phosphokinase-MB) assays were ordered by the assistant physicians, for clinical judgment. These results were presented as well.

The definition of SARS was in accordance with the Center for Disease Control and Prevention (CDC), which defines it as a severe acute respiratory syndrome associated with the diagnosis of SARS-CoV-2 infection. The definition of MIS-C was also in accordance with the CDC, which defines it as a severe illness leading to hospitalization in patients under 21 years of age, with a fever for at least 24 hours, showing laboratory evidence of inflammation, with multisystemic (≥ 2) organ involvement, confirmed or presumed SARS-CoV-2 infection, and no alternative plausible diagnosis.^18^

### Statistical analysis

For the assessment of data normality, the Kolmogorov-Smirnov test was performed to calculate the area under a normal curve, presumed as being when approximately 95% of the area was within 1.96 standard deviations of the mean. Continuous variables were expressed as mean ± standard deviation (SD) or median and interquartile range, according to the existence of normal data. Categorical variables were described as percentages. The Mann-Whitney test was used to compare continuous variables with skewed distribution. For the comparison of continuous variables with normal distribution, the unpaired student´s t test was used. The Kruskal-Wallis test, with the Conover-Iman method was used to compare VIS variances among patients with distinct combinations of findings on exams. The positive and negative predictive values of cTnI for the detection of 2D-TTE abnormalities were calculated. Two-sided p < 0.05 was considered statistically significant. All analyses were performed using StatsDirect, v. 3.3.4 2020 (Merseyside, UK).

## Results

Thirty-three patients were included in the study, with an age range of 31 days to 17 years. When the last patient was included, our study population represented 3% of all children diagnosed with COVID-19 in our region, meaning that nearly all critically ill children in our area must have been included in the study.^19^ The great majority (72.7%) presented a diagnosis of SARS. Three patients presented congenital heart diseases: one in status post-Senning operation (atrial baffle for D-transposition of the great arteries), another with a complete atrioventricular septal defect, and another one with an atrial septal defect, diagnosed during the study. There was one death due to a periorbital cellulitis complication in a patient with bone marrow aplasia. This patient had presented a diagnosis of SARS. Table 1 summarizes the general findings of our study.

**Table 1:** – Demographic and general features of paediatric patients with COVID-19

Characteristics	All patients (33)	SARS (24)	MIS-C (9)	p	95% CI
Male	19 (57.6%)	15 (62.5%)	4 (44.4%)	0.35	
Age (years)	6.4 ± 5.6	5.7 ± 5.6	8.2 ± 5.5	0.2	
BMI (Kg/m2)	18.2 ± 6	17.9 ± 5.8	18.9 ± 6.7	0.79	
**Chronic disorders**	**18 (54.5%)**	**16 (66.7%)**	**2 (22.2%)**	**0.02**	**0.06 to 0.69**
Haematologic	6 (18.2%)	6 (25%)	0		
Cardiac	3 (9.1%)	2 (8.3%)	1 (11.1%)		
Neurologic	3 (9.1%)	2 (8.3%)	0		
Obesity	3 (9.1%)	2 (8.3%)	1 (11.1%)		
Nephrologic	2 (6.1%)	2 (8.3%)	0		
Pulmonary	2 (6.1%)	2 (8.3%)	0		
Diabetes	1 (3%)	1 (4.2%)	0		
**Length of ICU stay (days)**	**6 (3-12)**	**7 (3-12)**	**5 (3-7)**	**0.32**	
Length of hospital stay (days)	14 (10-19)	15.5 (12-21.2)	10 (7-14)	0.09	
**Respiratory support**	**27 (81.8%)**	**22 (91.7%)**	**5 (55.6%)**	**0.008**	**0.05 to 0.67**
Oxygen Only	14 (42.4%)	13 (54.2%)	1 (11.1%)		
CPAP	1 (3%)	1 (4.2%)	0		
Mechanical ventilation	12 (36.4%)	8 (33.3%)	4 (44.4%)		
**Duration of respiratory support (days)**	**5 (1-9)**	**6(2.5-10.5)**	**2 (0-5)**	**0.14**	

*BMI: body mass index; ICU: intensive care unit; CPAP: continuous positive airway pressure.*

### Cardiac injury and cardiovascular compromise

Seventeen out of 33 patients (51.5%) presented with elevated cTnI and/or abnormal 2D-TTE. Five patients presented raised cTnI and abnormal 2D-TTE (15.2%); isolated raised cTnI was found in seven patients (21.2%); and an isolated abnormal 2D-TTE was found in five patients (15.2%).

Twelve patients (36.4%) needed cardiovascular support with inotropic or vasoactive drugs, chosen and titrated according to their clinical hemodynamic findings and at the discretion of the PICU team. Ten out of these 12 patients (83.3%) had abnormal cTnI and/or 2D-TTE, while among the 21 patients that did not need cardiovascular support, seven (33.3%) presented abnormal exams; this difference was statistically significant (p = 0.006, 95% CI = 0.15-0.73).

Among the five patients with both raised cTnI and abnormal 2D-TTE (a), 80% needed inotropic or vasoactive drugs (VIS 26 ± 24.8); among the patients with and isolated abnormal 2D-TTE (b), 60% needed these drugs (VIS 5 ± 5); among those with an isolated raised cTnI (c), 42.8% needed inotropic or vasoactive drugs (VIS 8.4 ± 13.4), whereas the use of these drugs was found in 12.5% of those patients with normal exams (d) (VIS 1.2 ± 3.6). Concerning VIS, a statistically significant difference was found only between patients “a” and “d” (p= 0.006); the difference between other pairwise comparisons were not significant (a vs b, p= 0.23; a vs c, p= 0.14; b vs c, p= 0.83; b vs d, p= 0.15; c vs d, p= 0.18). Table 2 summarizes the cardiovascular-related findings of our population.

**Table 2:** – Cardiovascular support and cardiac findings of paediatric patients with COVID-19

Characteristics	All patients (33)	SARS (24)	MIS-C (9)	p	95% CI
Cardiovascular support	12 (36.4%)	6 (24%)	6 (66.7%)	0.03	-0.69 to -0.04
Maximum VIS *	19 ± 17	11 ± 6	28 ± 21	0.051	-37.6 to 2.6
**Abnormal cTnI**	**12 (36.4%)**	**5 (20.8%)**	**7 (77.8%)**	**0.002**	**-0.79 to -0.19**
Peak cTnI		0.41 ± 0.48	0.61 ± 0.61	0.56	
Abnormal Echo and cTnI		1/5 (20%)	4/7 (57.1%)	0.2	
High pro-BNP	22/25 (88%)	15/17 (88.2%)	7/8 (87.5%)	0.96	
High CKMB	10/19 (52.6%)	5/11 (45.4%)	5/8 (62.5%)	0.46	
**Abnormal 2D-TTE**	**8 (24.2%)**	**4 (16%)**	**4 (44.4%)**	**0.08**	
LV systolic dysfunction	2 (6.1%)	0	2 (22.2%)	0.1	
RV systolic dysfunction	1 (3%)	1 (4.2%)	0	0.28	
TR/MR	5 (15.2%)	2 (8.3%)	3 (33.3%)	0.47	
Pericardial effusion	5 (15.2%)	4 (16.7%)	1 (11.1%)	0.03	0.04 to 0.96
WMA	1 (3%)	0	1 (11.1%)	0.28	
Coronary abnormality	0	0	0		
MLCA Z-score	-0.23 ± 0.8	-0.23 ± 0.88	-0.23 ± 0.57	1	
RCA Z-score	0.16 ± 0.87	0.14 ± 0.88	0.22 ± 0.9	0.81	

2D-TTE: two-dimensional transthoracic echocardiogram.; BNP: brain natriuretic peptide; CKMB: creatine phosphokinase-MB; cTnI: cardiac troponin I; VIS: vasoinotropic score; LV: left ventricular; MLCA: main left coronary artery; MR: mitral regurgitation; RCA: right coronary artery; RV: right ventricular; TR: tricuspid regurgitation; WMA: wall motion abnormality. *Of those who needed cardiovascular support.

2D-TTE abnormalities were found in 10 patients (30.3%). The most common findings were mild pericardial effusion (5 patients) and non-trivial mitral/tricuspid regurgitation (5 patients). Only two patients presented LV systolic dysfunction, who had fully recovered upon hospital discharge. One of the patients with LV systolic dysfunction also presented wall motion abnormality and a mild pericardial effusion. This patient had a cardiac magnetic resonance (CMR) performed 7 months later, and the pericardial effusion was still present, with no other CMR signs of myocarditis. No cases of coronary dilatation or pulmonary hypertension were found. Table 3 describes the main characteristics of the 10 patients that presented an abnormal 2D-TTE. Patient number 3, a boy in post-Senning operation status, presented SARS and atrial flutter. This patient had undergone a 2D-TTE at another institution 50 days ahead of the diagnosis of COVID-19. On that occasion, both tricuspid regurgitation and RV systolic dysfunction were considered mild, different from what was seen in this admission.

**Table 3:** – Clinical, laboratory and echocardiographic findings of the eight patients who presented an abnormal 2D-TTE upon admission

Patient number	1	2	3	4	5	6	7	8	9	10
Gender	M	M	M	F	F	F	M	F	F	F
Age	14 y	12 y	7 y	13 y	3 y	11 y	20 m	10 m	2 y	13 y
BSA	1.21	1.39	1.02	1.55	0.54	1.15	0.58	0.41	0.35	1.58
BMI	13.1	17.2	17.7	22.6	12.3	15.5	14.7	16.7	16.6	17.7
Comorbidity	SCD	-	CHD	-	BMA	-	-	-	CPD and CP	Diabetes
Presentation	SARS	MIS-C	SARS	MIS-C	SARS	MIS-C	MIS-C	SARS	SARS	SARS
Length of ICU stay W(days)	12	5	22	7	8	6	3	2	12	5
Length of hospital stay (days)	16	10	34	9	8	10	7	15	18	8
Respiratory support (days)	4	5	10	2	8	3	0	2	17	5
Mechanical ventilation (days)	0	3	2	0	1	2	0	0	0	2
Peak cTnI (cut-off 0.1 ng/ml)	< 0.1	0.27	< 0.1	1.91	< 0.1	0.58	0.22	0.13	< 0.1	< 0.1
cTnI normalization	-	3 days	-	4 days	-	5 days	1 day	1 day	-	-
Maximum VIS	0	50	5	15	10	55	10	0	10	0
Pro-BNP (cut-off 125 pg/ml)	NA	27183	5546	5341	988	22985	31188	1133	2445	NA
CKMB (cut-off 25 ng/ml)	NA	19.4	17.9	28.4	NA	41	23.6	24.4	NA	NA
Initial TTE	Minimum PE	Mild MR	Severe RV dysfunction, mild/moderate LV dysfunction, severe TR	Mild LV dysfunction, wall motion abnormality, mild PE	Mild PE	Moderate MR, mild LV dysfunction	Mild TR	Moderate PE	Mild PE	Mild MR
Discharge TTE	NA	normal	Severe RV dysfunction, mild LV dysfunction, moderate TR	Normal	Mild PE		Mild TR	Mild MR, minimum PE	NA	NA

*BMA: bone marrow aplasia; BNP: brain natriuretic peptide; CHD: congenital heart disease; CKMB: creatine phosphokinase-MB; CP: cerebral palsy; CPD: chronic pulmonary disease; LV: left ventricular; MR: mitral regurgitation; NA: not available; PE: pericardial effusion; RV: right ventricular; SCD: sickle cell disease; VIS: vasoactive inotropic score.*

### MIS-C vs SARS

The need for cardiovascular support was more frequent in patients presenting MIS-C than in those presenting SARS (66.7% and 25%, respectively, p 0.03, 95% CI = -0.7 to -0.04). Among those that needed cardiovascular support, the VIS score was higher in MIS-C patients than in SARS patients, but this finding was not statistically significant (28.2 ± 21.3 for MIS-C and 10.7 ± 5.7 for SARS, p= 0.1).

A raised cTnI was more frequently observed in patients with MIS-C than in patients with SARS (77.8% and 20.8%, respectively; p= 0.002, 95% CI = 0.19 to 0.79); however, the difference in peak cTnI was not statistically significant (p= 0.19). 2D-TTE abnormalities were found in 44.4% of the patients with MIS-C and in 25% of the patients with SARS (p= 0.28). The only statistically significant difference in the rate of 2D-TTE abnormalities were in the rate of LV systolic dysfunction, which was more common in MIS-C patients than in SARS patients (22.2% vs zero; p= 0.02, 95% CI = -0.06 to 0.55). No differences were found concerning length of ICU stay (p= 0.58), length of hospital stay (p= 0.86) and length of respiratory support (p= 0.61).

The positive predictive values of cTnI for the detection of 2D-TTE abnormalities were 70% for MIS-C patients and 20% for SARS patients. The negative predictive values were, respectively, 100% and 73.7%. Pro-BNP assay was not performed in all patients, since it was not part of the protocol, but an elevated pro-BNP was a common finding when performed (91.7%), regardless of the type of COVID presented by the patient. CKMB assay was not performed in all patients, since it was not part of the protocol, but it was elevated in 52.6% of the patients, with no significant difference according to COVID-19 presentation.

## Discussion

This study was developed with the aim of detecting signs of myocardial injury in critically ill paediatric patients, and to compare cardiac involvement between children presenting SARS and children presenting MIS-C. The diagnostic methods chosen for this assessment were transthoracic echocardiography and cardiac troponin I assay. Our PICU was the only referral unit for paediatric cases offered by the Brazilian Unified Health System (SUS, in Portuguese), in our region. Since the study was designed before the admission of the first case, it was possible to include all critically ill children with COVID-19 in our geographic area during the first COVID-19 wave in Brazil in 2020. This is possibly the most important strength of our study.

Myocardial involvement in COVID-19 is common and appears histologically in different forms: myocarditis-like disease, myocardial inflammation, thromboembolic disease and infarction. These findings have been supported by CMR imaging studies of adult and paediatric patients and by pathological evidence.^8 , 20 - 24^ However, these methods to diagnose myocarditis, myocardial edema or ischemic heart injury are not feasible in most of the children, due to the invasive nature of endomyocardial biopsy and to the difficulties in performing CMR imaging in acutely ill children, especially when there are constraints in using advanced imaging techniques during the COVID-19 pandemic.

cTnI is a cardiac-specific contractile protein found in cardiomyocytes and has a high sensitivity (95%) for the diagnosis of viral myocarditis in children.^25^ However, it can also be released in cases of excessive wall stress, myocardial ischemia or increased myocardial oxygen demand, situations often found in patients with COVID-19, especially in those with chronic medical conditions.^26^ One of the caveats about measuring cTnI in children is that the cut-off values are designed to diagnose infarctions in adults and these cut-offs may well be related to the amount of damaged tissue.^27^ Therefore, it is reasonable to argue that, if we are using adult cut-off values, the detection of raised cardiac troponin in children might be revealing a more extensive damage to the heart. Even in adults with COVID-19, abnormal CMR studies have been found without a simultaneous elevation in cardiac troponin.^20^ Nevertheless, some authors have reported their experience with MIS-C patients and have shown that troponin elevation is a common finding, and that it occurs in more than 70% of the cases.^6 , 7 , 28^ In our study, approximately 50% of the patients presented raised cTnI and/or echocardiographic abnormalities, confirming the high rate of myocardial injury in this subset of patients.

A very relevant finding in our work was the fact that patients who had both raised cTnI and abnormal 2D-TTE needed more cardiovascular support than those with normal exams, which is in agreement with some studies in the adult population with COVID-19 and SARS. These studies describe that the clinical course is worse when there is a troponin leak and an abnormal 2D-TTE.^29 - 31^ In our cohort, this was represented by a higher rate of patients in need of inotropic or vasoactive drugs, as well as in higher doses.

2D-TTE abnormalities were found in 30.3% of our population. The most common findings were mild pericardial effusion and mild mitral/tricuspid regurgitation. Only two patients presented LV systolic dysfunction, who fully recovered upon hospital discharge. The 2D-TTE abnormalities found in our study were mostly transitory and followed by a normalization of cTnI, revealing the dynamic course of the disease and, possibly, a healing process.

The two patients with LV systolic dysfunction represented only 16.7% of the patients who needed inotropes or vasopressors in our cohort, suggesting a major vasoplegic or inflammatory nature of this disease, as opposed to a state of low cardiac output syndrome. One of these patients with LV systolic dysfunction had the highest peak of cTnI in our cohort. Concerning LV systolic function, different findings have been described elsewhere: Grimaud and Ramcharam found a fall in LV ejection fraction in more than 80% of their MIS-C patients admitted to their PICUs presenting a shock.^6 , 32^ These differences might be related to local policies of ICU admission; different timing of diagnosis; different patient characteristics (demographics, presence of comorbidities); different patient genetic backgrounds with different interactions with SARS-COV2 causing distinct immune responses; different virus strains causing varying degrees of cardiovascular compromise; and different study methodologies (timing of imaging assessment, choice of method to assess cardiac function, etc.).

Raised cTnI was found in approximately 80% of the MIS-C patients and approximately 20% of the SARS patients; however, a raised cTnI was neither associated with LV systolic dysfunction, nor with circulatory shock. Five of the seven patients (71.4%) with raised cTnI in the MIS-C subgroup had some 2D-TTE abnormality, while in the subgroup with SARS, this rate was 20% (1/5). It is important to note that, in our study population, a normal cTnI assay upon admission had a very high negative predictive value, suggesting that this assay could be used to rule-out cardiac injury, avoiding unnecessary cardiovascular imaging in selected patients. While the number of patients with elevated cTnI is small, it still seems that there is a potential benefit of the cTnI assay as a screening test for 2D-TTE abnormalities. Furthermore, it was found that cTnI elevation and abnormal findings in 2D-TTE were transitory, behaving clinically as a usual non-fulminant acute myocarditis. In this context, timing for diagnosis is crucial, although the clinical impact of this diagnosis remains unknown. As for typical myocarditis, the incidence of chronic cardiomyopathy in the future of these children is unknown.

None of our patients presented coronary abnormalities, which is in agreement with findings from Grimaud et al, in France.^32^ Other studies also found a low rate of coronary abnormalities.^7 , 33^ Other imaging findings of COVID-19 in children have also been described. In the study by Ramcharan et al., 67% of the patients presented transient valve regurgitation.^6^ In the study by Grimaud et al., 65% of the patients presented non-trivial mitral or tricuspid regurgitation, and pericardial effusion was observed in 40% of their population.^32^ Non-trivial mitral or tricuspid regurgitation was found in 15% of our cohort, the same rate of pericardial effusion, with no difference between SARS and MIS-C.

Some cohorts have also reported changes in the heart rhythm in patients with systemic manifestations of SARS-CoV2 infection. In a New York cohort, with 393 patients, it was observed that 17.7% of patients hospitalized with SARS-COV2 and under mechanical ventilation, had atrial arrhythmias, versus 1.9% of those who did not need mechanical ventilation.^34^ In another cohort in China, with 187 patients, it was observed that 5.9% of the patients had tachyarrhythmias while they were hospitalized.^29^ The only case of arrhythmia seen in our study was in a child in post Senning operation status, a situation where atrial arrhythmias are known as a late complication of the surgery. In this case, it is unclear if the arrhythmia was triggered by COVID-19. This patient presented a worsening in his RV systolic function, with a normal cTnI.

### Study limitations

This study was designed in May, almost simultaneously with the announcement of the MIS-C phenotype of COVID-19. Therefore, we did not have enough information about MIS-C at that moment, and the design of the study did not include the assessment of inflammatory or coagulopathy markers, although some patients had done this type of blood work. The clinical haemodynamics (blood pressure, capillary refill time, heart rate, blood lactate) of the patients was not reviewed and the use of vasoactive or inotropic drugs was performed at the discretion of the assistant physicians. The present study opted to use the VIS score to represent the severity of the cardiovascular compromise in a standard fashion. Parameters of myocardial deformation were not evaluated, and we recognize that changes in strain may be present before the drop in the ejection fraction.

## Conclusions

The prevalence of signs of myocardial injury in COVID-19 infected children in need of intensive care was high (50%), and this was not exclusive of MIS-C patients. MIS-C patients with both elevated cardiac troponin I and abnormal findings in the 2D-TTE very often present signs of shock. Markers of cardiac injury were transitory and early outcomes, in general, were favorable. Finally, considering the high number of infected patients recently admitted to PICUs around the world and that health resources may be limited, performing a cTnI assay might help healthcare providers to discriminate those patients with a more urgent need for 2D-TTE.
